# The relationship between the Mediterranean diet and Axis I disorders: A systematic review of observational studies

**DOI:** 10.1002/fsn3.2950

**Published:** 2022-06-06

**Authors:** Samaneh Madani, Afsane Ahmadi, Firoozeh Shoaei‐Jouneghani, Mahsa Moazen, Najmeh Sasani

**Affiliations:** ^1^ Nutrition Research Center School of Nutrition and Food Sciences, Shiraz University of Medical Sciences Shiraz Iran

**Keywords:** anxiety, axis I disorders, depression, eating disorders, Mediterranean diet, systematic review

## Abstract

Axis I disorders are one of the major health burdens worldwide. Evidence suggests that Mediterranean diet has key biological factors associated with reducing the progression of these disorders. This systematic review aimed to clarify the relationship between Mediterranean diet and Axis I disorders. PubMed and Scopus databases were searched from January 2016 up to June 2021. Those observational studies in English language that assessed the relationship between Mediterranean diet and Axis I disorders (such as depression, anxiety, eating disorders, schizophrenia, etc.) were included in this review. The Newcastle–Ottawa Scale was used to evaluate the quality of studies. Thirty‐six studies (15 cohorts, 19 cross‐sectional, and 2 case–control) met the inclusion criteria. The results revealed that more than two‐thirds of the studies (25 studies, 69.44%) had significant protective relationship between receiving Mediterranean diet and reducing the symptoms or incidence of Axis I disorders. Most studies were performed on depression (29 studies measured depression at least as one of the Axis I disorders), of which 72.41% reported an inverse relationship. There were also 9 studies on anxiety (studies that measured anxiety at least as one of the Axis I disorders), that 77.77% of them observed protective association. Moreover, majority of the studies (25 studies, 69.44%) had high quality, of which 76% found an inverse relationship. In conclusion, it seems that the Mediterranean diet can reduce the symptoms or the occurrence of Axis I disorders (especially depression and anxiety). However, more extensive review studies, particularly with interventional designs, are necessary to prove the result.

## Graphical abstract

This systematic review aimed to clarify the relationship between Mediterranean diet and axis I disorders in adults. Evidence suggests that Mediterranean diet have key biological factors associated with reducing the progression of these disorders. The results of this systematic review revealed that more than two‐thirds of the included studies had significant protective relationship between receiving Mediterranean diet and reducing the symptoms or incidence of Axis I disorders.

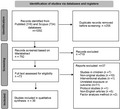

## 1 INTRODUCTION

Axis I disorders are a group of clinical psychopathological disorders that include depression, anxiety, bipolar disorders, schizophrenia and psychosis, eating disorders, obsessive–compulsive disorders, trauma and stress‐related disorders, and substance abuse (Bagby et al., 2017). Attention‐deficit/hyperactivity disorder (ADHD) can also be classified as an Axis I disorder based on the diagnostic and statistical manual of mental disorders‐IV (DSM‐IV) system (Haavik et al., 2010). Today, mental disorders have become a major global health problem, with its prevalence increasing sharply even in young people. Inappropriate diet, smoking, and inadequate physical activity are some of the causes of mental disorders, particularly anxiety and depression (Firth et al., 2020).

Depression is one of the most important mental disorders in different ages (especially in adulthood and elderly) and is a basis for other diseases that can have devastating effects on mental health and quality of life (Casey, 2017; Lai et al., 2017). According to the reports of World Health Organization (WHO), this disorder is expected to be one of the most common mental disorders in communities in the 2030s (Gianfredi et al., 2021). Anxiety is another common Axis I disorder that can lead to mental and functional problems as well as gastrointestinal problems, diabetes, and thyroid disorders (Masana et al., 2019). Another mental disorder that affects societies is bipolar disorder, which is less common than depression and anxiety, but is more severe and chronic. This mental disorder can impair job performance and lead to suicide (Khan et al., 2019). Schizophrenia is also a mental problem with certain complications and can increase the mortality rate by 2 to 3 times compared to the general population and put people at increased risk of cardiovascular diseases, cancer, metabolic syndrome, etc. (Charlson et al., 2018; Costa et al., 2018; Costa et al., 2019). Eating disorders, which fall into the category of Axis I disorders as well, are characterized by abnormal eating and dissatisfaction with weight and body shape (Leone et al., 2018). Besides, obsessive–compulsive disorder causes significant changes in social and occupational functioning (Ince et al., 2017). In addition to these disorders, ADHD is one of the most common neuroviral disorders in childhood and often persists into adulthood. This feature is characterized by difficulty in maintaining attention to daily activities (San Mauro Martín et al., 2018). Post‐traumatic stress disorder is also a debilitating mental disorder that occurs in a subset of people after a major traumatic event. The relevant fear can be easily activated in these people even in the absence of threats (Hori & Kim, 2019).

Considering the increasing incidence of these disorders and the heavy financial burden they impose on individuals and the public health system, preventive, rapid, and low‐cost strategies to reduce the relevant consequences are essential (Lai et al., 2017). Various factors can play an important role in controlling Axis I disorders, including appropriate diet, adequate physical activity, and medications. A number of studies in recent years have shown the effective and significant role of the Mediterranean diet in the prevention and controlling of Axis I disorders (Açik et al., 2020; Recchia et al., 2020; Vall Castelló & Tubianosa, 2020). Nevertheless, in some investigations no relationship has been found between this dietary pattern and Axis I disorders (Cherian et al., 2021; Hernández‐Galiot & Goñi, 2017; Masana et al., 2019).

The Mediterranean diet refers not only to the type of food consumed, but also to the lifestyle and social customs associated with the way individuals eat (Hernández‐Galiot & Goñi, 2017). This diet, first described by Keys Ancel, is characterized by a high consumption of fruits, vegetables, unrefined cereals, olive oil, nuts, and seafood; moderate consumption of chicken, dairy products, and red wine; and low consumption of red meat. Adherence to this diet is associated with a reduced risk of several chronic diseases, especially cardiovascular diseases, various types of cancer, and type 2 diabetes mellitus (Mantzorou et al., 2021). The Mediterranean diet can also play a protective role against psychological disorders due to its high content of fiber, omega‐3 fatty acids, vitamins B, E, magnesium, antioxidants, and phytoestrogens (Sadeghi et al., 2021). Moreover, several important components in this diet such as legumes, nuts, and fish are important factors in controlling and reducing the process of nerve damage (Hernández‐Galiot & Goñi, 2017).

With regard to the prevalence of mental disorders and its association with consuming Mediterranean diet, the prevalence of depression in Spain, where Mediterranean diet is generally consumed (Moreiras‐Varela, 1989), was reported to be 4.73% in 2015–2017. Based on the European Study of the Epidemiology of Mental Disorders (ESEMeD), the prevalence in Spain is lower than other countries in Europe (Vieta et al., 2021). For instance, the prevalence of depressive disorders in Germany was equal to 15.7% in 2017 (Steffen et al., 2020). It has been found that plant foods are consumed in lower amounts by Germans compared to Mediterranean residents, while the consumption of animal products is higher (Leonhäuser et al., 2004).

Therefore, due to the importance of the issue and the lack of consistency between the results obtained from primary studies, this systematic review, for the first time, aimed to evaluate the relationship between the Mediterranean diet and Axis I disorders. Furthermore, in this review, those Mediterranean diet scores that were determined by priori approach were considered, not those Mediterranean dietary patterns that were derived from factor analysis, or in other words from posteriori approach.

## 2 METHODS

The Preferred Reporting Items for Systematic Reviews and Meta‐analyses (PRISMA) guideline was used for conducting this systematic review (Moher et al., 2009).

### 2.1 Search strategy and study selection

PubMed and SCOPUS online databases were used to search for published articles. The time span of the literature search was a 5‐year period from January 1, 2016 to June 18, 2021. The search strategy was developed according to medical subject heading (MeSH) terms. General terms used in the search strategy included depression, bipolar disorder, anxiety, schizophrenia, psychosis, obsessive–compulsive symptom, attention‐deficit/hyperactivity disorder, post‐traumatic stress disorder, eating disorders (including anorexia nervosa, bulimia nervosa, and binge eating disorder), and Mediterranean diet. The complete search strategy is available in Appendix A.

Those observational studies (cohort, cross‐sectional, and case–control studies) that were published in English language and had available full‐texts were included in the present systematic review. Besides, those studies that were conducted in children or adolescents (age <18 years), or derived the Mediterranean dietary pattern from factor analysis (a statistical method that is different from direct scoring), were excluded from this review.

PECOS were as follows: Participants: all individuals, except children and adolescents; Exposure: Mediterranean diet; Control group: any control group; Outcomes: Axis I disorders including depression, bipolar disorder, anxiety, schizophrenia, psychosis, obsessive–compulsive symptom, attention‐deficit/hyperactivity disorder, post‐traumatic stress disorder, and eating disorders (including anorexia nervosa, bulimia nervosa, and binge eating disorder); Study design: cohort, cross‐sectional, and case–control studies.

The retrieved studies were screened by titles and abstracts for eligibility. Afterwards, full‐texts of the articles that did not provide enough information according to title and abstract were assessed by two independent reviewers and discrepancies were resolved through discussion.

### 2.2 Data extraction

Two authors independently extracted data from the articles and discussed in case of disagreement. This information included name of the first author, date of publication, the country in which the study was conducted, the characteristics of the participants, gender, age, sample size, method of assessing the exposure (Mediterranean diet), method of assessing the outcome, follow‐up duration (for cohort studies), and the results obtained. Most of these studies used measures of associations (such as odds ratio or hazard ratio) to explore the relationship between Mediterranean diet and Axis I disorders.

### 2.3 Quality assessment

The Newcastle–Ottawa Scale (NOS) was used to evaluate the overall quality of studies (cohort, cross‐sectional, and case–control studies). The NOS checklist consists of selection, comparability, and exposure/outcome sections. Each section was assigned maximum of four, two, and three points, respectively, for case–control or cohort studies; and five, two, and three points, respectively, for cross‐sectional studies. According to NOS thresholds, the studies were categorized into good, fair, and poor quality (Stang, 2010). The quality assessment was performed independently by two authors and disagreements were resolved by consensus.

## 3 RESULTS

**FIGURE 1:**
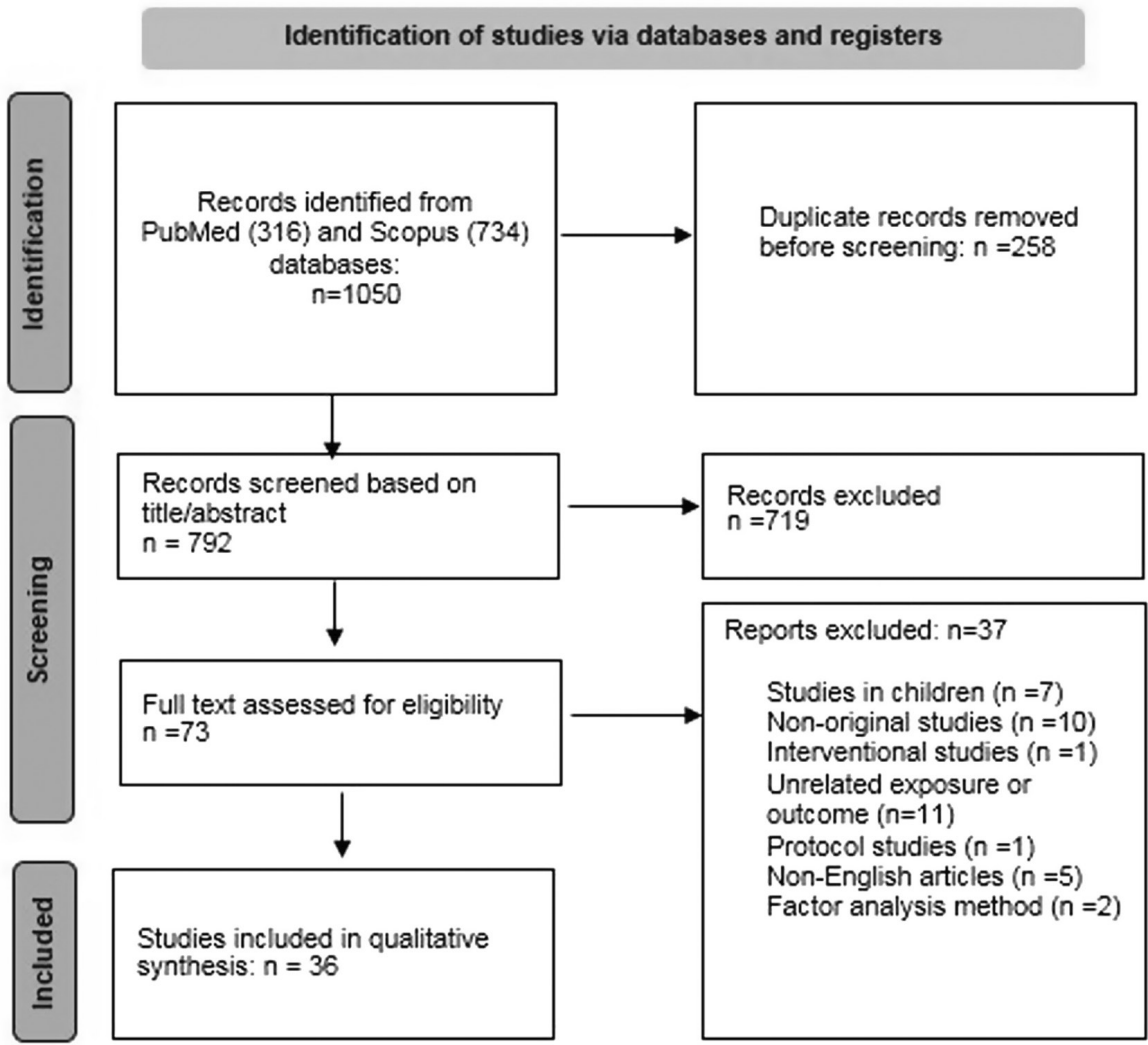
Flow diagram of the literature search process and selection of articles

A total of 1050 articles were found in initial search, leaving 792 articles after removing duplicates (258 items). After screening by title and abstract, another 719 articles were deleted and 73 articles remained for full‐text evaluation. Out of 73 articles, 36 studies had inclusion criteria and 37 other articles were excluded for the following reasons: 7 articles due to research on children or adolescents, 5 articles due to non‐English language, 2 articles due to the use of factor analysis method, 11 articles due to the lack of evaluating the relevant exposure or outcome, 10 articles due to not being original, 1 article due to being protocol study, and 1 article due to having interventional design. The selection procedure of the studies can be found in Figure 1.

### 3.1 Characteristics of studies

Tables 1 and 2 summarize the study characteristics of the included articles. Of the 36 articles included in this systematic review, 15 were cohort, 19 were cross‐sectional, and 2 were a case–control study. Of these articles, most of them (29 studies, 80.56%) were conducted in Europe. In terms of gender, most studies (29 studies, 80.56%) were performed on both sexes and the mean age of the participants varied from 21.42 to 92.7 years. The sample size range for the cohort studies was 273–167,698, for the cross‐sectional studies was 79–4470, and for the case–control studies was 141–273.

**TABLE 1:** Summary of cohort studies exploring the association between the Mediterranean diet and Axis I disorders

	Study	Country	Subject characteristics/gender/age (baseline)/sample size	Method of exposure measurement	Outcome(s)/method of outcome(s) measurement	Follow‐ up duration	Results	Quality
1	Cherian et al. (2021)	USA	Participants living in retirement communities and senior public housing units/Both sexes/80.4/*n* = 709	FFQ/MDS/11 items/range 0–55	Depression/CES‐D/10‐item	6.53 years	No significant differences were observed between tertiles of MDS and incidence of depressive symptoms.	Good
2	Gianfredi et al. (2021)	Netherlands	A population enriched with T2DM/Female/59.9/*n* = 2646	FFQ/MDS/9 items/range 0–9	Depression/PHQ‐9/9 items	6.1 years	MD was not significantly associated with incident depressive symptoms.	Fair
3	Das et al. (2021)	Australia	Participants aged ≥70 years/Male/81.1/*n* = 781	Standardized diet history questionnaire/MDS/used as a continuous variable	Depression/GDS/15 items	3 years	There was no significant association between MD and incident GDS≥5	Poor
4	Vall Castelló and Tubianosa (2020)	27 European countries	Elderly individuals/female/64.99/*n* = 167,698	Survey questions/Mediterranean diet/4 items	Depression/EURO‐D scale included in the SHARE questionnaire/12 items/range 0–12	6 years	A significant relationship was found between receiving MD and reducing depressive symptoms	Good
5	Recchia et al. (2020)	London	Adult participants from the Whitehall II study/both sexes/35–55/*n* = 4949	FFQ/tMDS/9 items/range 0–9	Depression/CES‐D/20 items	13 years	Higher tMDS score was associated with lower risk of recurrent depressive symptoms	Good
6	Winkens et al. (2020)	Netherlands	Dutch older adults/both sexes/66.7/*n* = 929	FFQ/MDS/11 items/range 0–55	Depression/CES‐D/20 items	3 years	Significant negative correlation was found between MDS and depressive symptoms.	Poor
7	Fresán et al. (2019)	Spain	University graduates/both sexes/34/*n* = 15,980	FFQ/MDS/9 items/range 0–9	Depression/Being diagnosed with depression by a medical doctor or report of regular use of antidepressant medication	10.4 years	Increased adherence to the Mediterranean diet was inversely associated with depression risk	Good
8	Ruiz‐Estigarribia et al. (2019)	Spain	Spanish university graduates without depression or chronic prevalent diseases/Both sexes/36.7/*n* = 14,908	FFQ/MDS/8 items/range 0–8	Depression/Having been diagnosed by a medical doctor or habitual new use of antidepressant treatments.	10.4 years	A significant inverse association was observed for medium‐to‐high MDS adherence and risk of depression	Good
9	Elstgeest et al. )^2019^)	Netherlands	Participants in the LASA Nutrition and Food‐related Behaviour study (middle‐aged and older individuals)/both sexes/71.2/*n* = 687	FFQ/MDS*/11 items/range 0–55	Depression/CES‐D/20 items/range 0–60	14 years	Men with a long‐term depressive symptoms' history had lower MDS. (In cross‐sectional analyses, depressive symptoms were associated with lower MDS scores in men after adjustment for confounders.)	Good
10	Leone et al. (2018)	Spain	Previous students of the University of Navarra, Spanish registered professionals, and other university graduates/female/34.33/*n* = 11,800	FFQ/MDS/9 items/range 0–9	Anorexia or bulimia nervosa/Positively responding to the question of having been diagnosed with anorexia or bulimia nervosa by a physician	9.4 years	A lower anorexia or bulimia nervosa risk was observed for upper categories of adherence to MD.	Good
11	Adjibade et al. (2018)	France	Participants of the SU.VI.MAX study without depression at baseline/both sexes/49.5/*n* = 3523	24‐h dietary records/rMED/9 components/range 0–18	Depression/CES‐D/20 descriptive statements/range 0–60	12.6 years	Higher rMED score was significantly associated with lower incident depressive symptoms in men, but not in women	Good
12	García‐Toro et al. (2016)	Spain	Patients with major depressive disorder, with mild to moderate depressive symptoms for at least 2 months/Both sexes/51/*n* = 273	MEDAS/14‐item	Depression/Major depression: MINI Depression severity: BDI‐II	12 months	MEDAS was inversely associated with basal BDI. However, the longitudinal association was not significant.	Poor
13	Notara et al. (2016)	Greece	Acute coronary syndrome patients/both sexes/63.5/*n* = 2172	FFQ/MDS/11 items/range 0–55	Depression/CES‐D/range 0–60	10 years	Patients with the highest MDS were less likely to suffer from high depressive symptoms	Good
14	Sánchez‐Villegas et al. (2016)	Spain	Spanish university graduates who did not have depression and did not take antidepressants at baseline/both sexes/37.8/*n* = 11,800	FFQ/MDS/10 items/Participant obtained 0 to 100% adherence	Depression/Depression was defined as the habitual use of antidepressant drugs or self‐reported diagnosis of depression performed by a physician.	8.5 years	MD was inversely associated with depression risk.	Good
15	Lai et al. (2016)	Australia	Middle‐aged women/female/45–50/*n* = 9280	DQES v2/MDS/9 items (range 0–9)	Depression/CES‐D scale/10‐item	12 years	A significant inverse association was found between MD and depressive symptoms.	Good

*Note:* Ages are presented as mean or ranges, except for the studies of Kaufman‐Shriqui et al., 2021, Elstgeest et al., 2019, and Fresán et al., 2019 which were reported as median.

Abbreviations: BAI: Beck Anxiety Inventory, BDI‐II: Beck Depression Inventory, CES‐D: Center for Epidemiologic Studies Depression Scale, CIDI:Composite International Diagnostic Interview, CIDI‐SR: Inventory of Depressive Symptomatology‐Self Report, DQES v2: Dietary Questionnaire for Epidemiological Studies version 2, EURO‐D: European Union initiative to compare symptoms of Depression, FFQ: Food Frequency Questionnaire, GDS:Geriatric Depression Scale, MD: Mediterranean Diet, MDS: Mediterranean Diet Score, MEDAS: Mediterranean Diet Adherence Score, MINI: Mini International Neuropsychiatric Interview, NESDA: Netherlands Study of Depression and Anxiety, PHQ‐9: 9‐Item Patient Health Questionnaire, rMED: relative Mediterranean diet score, SHARE: Survey of Health, Ageing and Retirement in Europe, SU.VI.MAX: The Antioxidant Vitamins and Minerals Supplementation, tMDS: transformed Mediterranean Diet Score, T2DM: Type 2 Diabetes Mellitus.

**TABLE 2:** Summary of cross‐sectional and case–control^a^ studies exploring the association between the Mediterranean diet and Axis I disorders

	Study	Country	Subject characteristics/gender/age/sample size	Method of exposure measurement	Outcome(s)/method of outcome(s) measurement	Results	Quality
1	Mantzorou et al. (2021)	Greece	Elderly participants who were free of diseases/both sexes/74.97/*n* = 2092	MDS/Based on the median values, those with scores ≤25, 26–28, 29–31, and ≥32 were classified as very low, low, moderate, and high adherence, respectively	Depression/GDS	Higher MD adherence was strongly associated with less depressive symptoms.	Good
2	Vassou et al. (2021)	Greece	Participants with no clinical evidence of cardiovascular diseases, other atherosclerotic diseases, or chronic viral infections/both sexes/42.75/*n* = 853	FFQ/MDS/11 items/range 0–55	Anxiety/STAI/20 items Depression/ZDRS/range 20–80	MDS was inversely correlated with anxiety, but was positively correlated with depression.	Good
3	Riera‐Sampol et al. (2021)	Spain	Patients with cardiovascular risk factors from primary care centers/both sexes/62.2/*n* = 310	MDS/14‐item/range 0–14	Depression/PHQ‐9/range 0–20	Higher MDS was associated with lower depression levels	Good
4	Sadeghi et al. (2021)	Iran	General adults from the project of “Studying the Epidemiology of Psycho‐Alimentary Health and Nutrition”/both sexes/36.54/*n* = 3172	FFQ/MDS/9 items/range 0–9	Depression and anxiety/HADS/14 items/range 0–21	Inverse associations were found between adherence to MD and depression or anxiety.	Good
5	Kaufman‐Shriqui et al. (2021)	This was an international study	Adult individuals/both sexes/33/*n* = 3797	I‐MEDAS/14‐item/range 0–17	Anxiety/GAD‐7/7 items/range 0–21	Higher anxiety score was associated with lower Mediterranean diet score	Poor
6	Trigueros et al. (2020)	Spain	Students of the University of Almeria/both sexes/23.58/*n* = 1347	KIDMED/16 items/ranges 0–12	Anxiety/For exam anxiety, the Test Anxiety Inventory was applied.	Test anxiety was reversely related to MD.	Poor
7	Carlos et al. (2020)	Spain	University students without psychological diagnosis or drugs consumption/both sexes/21.42/*n* = 252	KIDMED/16 items	Anxiety/STAI/40 items	Only trait‐anxiety was a predictive variable for adherence to MD and a direct relationship was found.	Good
8	Açik et al. (2020)	Turkey	Participants who referred to the social facility of the Ankara Metropolitan Municipality./female/42.15/*n* = 977	24‐h dietary recall/PREDIMED/14 items/range 0–14	Depression and anxiety/DASS/42 items/score range of 0–42 on each subscale	Higher PREDIMED score was associated with lower odds of depression or anxiety.	Good
9	Gibson‐Smith et al. (2020)	Netherlands	Participants were selected in different regions from the general population, in general practice and in mental health organizations (78% of baseline sample had a lifetime depressive or anxiety disorder)/both sexes/52/*n* = 1634	FFQ/MDS/11 items/range 0–55	Depression/IDS‐SR/30‐item/range 0–84 Anxiety/BAI/21 items/range 0–63	The MDS had inverse significant relationships with depression and anxiety	Good
10	Vicinanza et al. (2020)	Italy	Geriatric medical outpatients/both sexes/73.11/*n* = 143	MDQ/14‐item/range 0–14	Depression/GDS/15‐item	Significant inverse association was found between the MDQ score and GDS.	Poor
11	Paans et al. (2019)	Netherlands	Healthy, remitted, and current patients from the Netherlands Study of Depression and Anxiety/both sexes/51.7/*n* = 1442	FFQ/MDS/11 items/range 0–55	Depression/CIDI and IDS‐SR (30‐item, range 0–84)	Depression and severity of it were associated with lower MDS.	Good
12	Mahdavi‐Roshan et al. (2019)	Iran	Adults with cardiovascular disease risk factors/both sexes/58.33/*n* = 344	MEDAS/14‐point	Depression/BDI/range 0–63	No relationship was discovered between depression and dietary adherence after controlling for confounders.	Good
13	Costa et al. (2019)	Portugal	Inpatients and outpatients with schizophrenia/both sexes/44.57/*n* = 100	FFQ/MDS/9 items/range 0–9	Schizophrenia/Diagnosis of schizophrenia was based on the DSM‐5 criteria.	No significant difference was observed in MDS between inpatients and outpatients.	Poor
14	Masana et al. (2019)	Greece	Older adults without preexisting cardiovascular disease or other chronic diseases/both sexes/59.67/*n* = 1128	FFQ/MDS/range 0–55	Anxiety/STAI/20‐item/range 20–80	No significant relationship was observed between MD and anxiety.	Good
15	Masana et al. (2018)	Mediterranean islands	Older people living in the Mediterranean basin./Both sexes/74.2/*n* = 2718	MDS/11 items/range 0–55	Depression/GDS/range 0–15	Participants with mild or severe depression had lower adherence to MD.	Good
16	Gibson‐Smith et al. (2018)	Netherlands	Participants were recruited from the general population, general practice, and mental health organizations (78% of baseline sample had a lifetime depressive or anxiety disorder)/both sexes/52/*n* = 1634	FFQ/MDS/11 items/range 0–55	Depression and anxiety/Presence of disorders by CIDI; Severity of depression by IDS‐SR/Severity of anxiety by BAI	Severity of depression or anxiety, or having both disorders currently was significantly associated with lower MDS.	Good
17	Pagliai et al. (2018)	Italy	Nonagenarians enrolled in the Mugello Study/both sexes/92.7/*n* = 388	MDS/11 items/range 0–55	Depression/GDS/15‐item	No significant differences were found for MDS between depressed and non‐depressed subjects.	Good
18	Hernández‐Galiot and Goñi (2017)	Spain	An elderly non‐institutionalized population/both sexes/81/*n* = 79	24‐h diet recall/MEDAS/14‐item/range 0–14	Depression/GDS/range 0–15	No relationship was found between the MEDAS and GDS.	Poor
19	Veronese et al. (2016)	North America	Patients with a high risk of knee osteoarthritis/both sexes/61.3/*n* = 4470	FFQ/MDS/11 items/range 0–55	Depression/CES‐D/20‐item/range 0–60	Higher MDS was associated with reduced depressive symptoms.	Good
20	Łojko et al. (2019)	Poland	Cases were patients treated for bipolar disorder for more than 5 years in the outpatient clinic. The control group was selected from local primary health services' users/both sexes/58.7/*n* = 273	FFQ/MDS/11 items/range 0–55		Bipolar patients had lower MDS compared to controls.	Poor
21	Della Camera et al. (2017)	Italy	Patients with erection disorder or without erection disorder referred to an andrology department/male/64.3/*n* = 141	MDQ/14 items	Depression/the Hamilton scale/21 items/range 0–23	Strong adherence to MD showed protective effect against depression.	Poor

*Note:* Ages are presented as mean.

Abbreviations: BAI: Beck Anxiety Inventory, BDI: Beck Depression Inventory, CES‐D: Center for Epidemiologic Studies Depression Scale, CIDI: Composite International Diagnostic Interview, DASS: Depression Anxiety Stress Scales, DSM‐5: Diagnostic and Statistical Manual of Mental Disorders, FFQ: Food Frequency Questionnaire, GDS: Geriatric Depression Scale, GAD‐7: 7‐item Generalized Anxiety Disorder Scale, HADS: Hospital Anxiety and Depression Scale, I‐MEDAS: Israeli Mediterranean diet screener, IDS‐SR: Inventory of Depressive Symptomatology‐Self Report, KIDMED: Mediterranean Diet Quality Index, LASA: Longitudinal Aging Study Amsterdam, MDS: Mediterranean Diet Score, MDQ: Med‐Diet Questionnaire, MEDAS: Mediterranean Diet Adherence Screener, MD: Mediterranean Diet, PHQ‐9: 9‐item Patient Health Questionnaire, PREDIMED: Prevention with Mediterranean Diet, STAI: State–Trait Anxiety Inventory, ZDRS: Zung Self‐Rating Depression Scale.

^a^ All the studies are cross‐sectional, except for the last two studies that had adopted case–control design.

Evaluation of the exposure variable (i.e., the Mediterranean diet) was mostly calculated by the 11‐item Mediterranean diet score (MDS; score range 0–55; 13 studies) and the 9‐item Mediterranean diet score (score range 0–9; 7 studies). Evaluation of the outcome variable, which included Axis I disorders, was also measured by specific instruments. For example, depression was assessed by scales such as the Center for Epidemiologic Studies Depression Scale (CES‐D; 8 studies), Geriatric Depression Scale (GDS; 6 studies), the 9‐item Patient Health Questionnaire (PHQ‐9; 2 studies), Inventory of Depressive Symptomatology‐Self Report (IDS‐SR; 2 studies), and Composite International Diagnostic Interview (CIDI; 2 studies). Besides, Spielberger State–Trait Anxiety Inventory (STAI) was one of the questionnaires used to assess anxiety (2 studies). The duration of cohort follow‐ups was also varied from 12 months to 14 years.

### 3.2 Findings

Out of 36 eligible studies, 25 articles (69.44%) found an inverse relationship) significant protective effect) between the Mediterranean diet and Axis I disorders, 9 studies (25%) showed no significant association, and 1 article (2.78%) showed a direct relationship (significant detrimental effect). Moreover, one study (2.78%), which evaluated anxiety and depression simultaneously, reported a significant protective relationship between anxiety and the Mediterranean diet and a significant direct association between depression and the Mediterranean diet.

In terms of study design, among the 15 cohort studies, 11 (73.33%) showed significant protective association and 4 (26.67%) showed nonsignificant relationship between the Mediterranean diet and Axis I disorders. Out of 19 cross‐sectional studies, 12 studies (63.16%) found significant protective relationship, 5 studies (26.32%) found nonsignificant results, 1 article (5.26%) showed a significant direct relationship between Mediterranean diet and trait‐anxiety, and 1 article (5.26%) showed a significant protective relationship between anxiety and the Mediterranean diet and a significant direct relationship between depression and the Mediterranean diet. The 2 case–control studies also showed significant protective relationship.

Out of 15 cohort studies, 14 studies examined the relationship between the Mediterranean diet and depression, and 1 study examined the relationship between this diet and eating disorders. Of the 14 cohort studies related to depression, 10 studies showed a significant protective relationship and 4 studies showed a nonsignificant relationship. In the one study that assessed eating disorders, protective relationship was observed.

Out of 19 cross‐sectional studies, 9 studies examined the relationship between the Mediterranean diet and depressive disorder, 5 studies examined the relationship between this diet and depression and anxiety, 4 studies examined the relationship between the Mediterranean diet and anxiety, and 1 study examined the association between the diet and schizophrenia. Out of 9 cross‐sectional studies related to depression, 6 showed a significant protective relationship and 3 found a nonsignificant association. Out of 4 cross‐sectional studies related to anxiety, 2 studies showed a significant protective association, 1 study showed a nonsignificant relationship, and 1 study showed a significant direct relationship. Of the 5 cross‐sectional studies on anxiety and depression, 4 showed significant protective associations between depression and anxiety, and 1 showed a significant protective relationship between anxiety and the Mediterranean diet and a significant direct relationship between depression and the Mediterranean diet. Moreover, the cross‐sectional study examining the association between the Mediterranean diet and schizophrenia reported a non‐significant relationship. Besides, one case–control study was conducted on bipolar patients and showed a significant protective relationship, and one case–control study was performed on depressive disorder and found a significant protective association.

Therefore, among all the studies that examined the relationship between the Mediterranean diet and depression, 72.41% showed a significant protective relationship, 24.14% reported a non‐significant association, and 3.45% found a significant direct relationship. Additionally, among the studies that examined the relationship between the Mediterranean diet and anxiety, 77.8% showed a significant protective relationship, 11.1% found a non‐significant association, and 11.1% reported a significant direct relationship. Furthermore, the only study on eating disorders and the only study on bipolar disorder reported significant protective relationships and the only study on schizophrenia showed a non‐significant association.

### 3.3 Quality of studies

Results from the quality assessment of the studies indicated that 25 studies (69.44%) had good quality, 10 studies (27.78%) had poor quality, and only 1 study (2.78%) had fair quality. Of the high‐quality studies, 19 (76%) articles showed significant protective relationships between the Mediterranean diet and axis I disorders, and 1 study found protective relationship with anxiety and direct relationship with depression. Of the cohort studies, 11 (73.33%) had good quality, that 10 (90.90%) of them found significant protective relationships. Moreover, of the cross‐sectional studies, 14 (73.7%) articles had high quality, of which 9 (64.3%) studies showed significant protective associations. The 2 case–control studies had poor quality. The detailed results are available in Appendix B.

## 4 DISCUSSION

Considering the high prevalence of mental disorders in modern societies, applying strategies to prevent serious psychological problems is crucial. Diets such as the Mediterranean diet may help to control the progression of mental disorders (Bastos et al., 2020). As a result, a systematic review was conducted to determine the relationship between the Mediterranean diet and Axis I disorders.

A careful review of observational studies conducted in this area showed that the Mediterranean diet can be effective in controlling or preventing Axis I disorders. Overall, out of 36 studies included in this review, most of them (25 studies, 69.44%) reported significant protective association. Moreover, most studies of the present review had been carried out on depression and the results of 72.41% of these studies showed an inverse association between the Mediterranean dietary pattern and the symptoms or prevalence of depression. Majority of studies on anxiety (77.8% of studies) also showed significant protective relationship. Most of the studies also had high quality (69.44%), that 76% of them reported significant protective association.

The Mediterranean diet can have protective effects on mental health due to the presence of B vitamins (B_1_, B_6_, B_9_, and B_12_) (Sánchez‐Villegas et al., 2006). This diet increases the body's antioxidant capacity by having plant‐based components rich of vitamins, flavonoids, minerals, and phenolic compounds (Ortega, 2006; Pitsavos et al., 2005). Oxidative stress leads to an increased inflammatory status through the activation of transcription factors and expression of pro‐inflammatory genes. It has been suggested that chronic mild inflammatory pathways in the periphery and the brain, which is called neuroinflammation, have a role in the development of depression and anxiety (Xu et al., 2014). Therefore, the presence of antioxidant compounds in the Mediterranean diet can be involved in the management of these disorders. Moreover, since it is recommended to consume more fruits, nuts, and vegetables in the diet, the intake of minerals especially magnesium will increase. Studies have shown a significant protective relationship between magnesium intake and the reduction of symptoms of mental disorders, especially depression (Rajizadeh, Mozaffari‐Khosravi, Yassini‐Ardakani, & Dehghani, 2017). The abundance of antioxidants and folic acid in the diet, due to high intakes of vegetables, can also reduce oxidative stress, improve mood, and control anxiety (Gibson‐Smith et al., 2020). Besides, children with ADHD generally eat less fruits, vegetables, and fatty fish, and are more likely to eat junk food, artificial food colorings, sugar, and fast food. Iron, zinc, copper, and omega‐3 deficiencies have also been reported in children with ADHD (Ríos‐Hernández et al., 2017; Woo et al., 2014). Studies have shown the positive effect of the Mediterranean diet on ADHD due to the modification of dietary pattern (Ríos‐Hernández et al., 2017). Those investigations performed on the effect of the Mediterranean diet on eating disorders have also shown that higher intakes of monounsaturated fatty acids (olive oil), polyunsaturated fatty acids (fish), and nuts can affect the brain serotonin pathway and thus can be effective in eating disorders (Bertoli et al., 2015). Additionally, some components of the Mediterranean diet such as increased monounsaturated fatty acids' intake compared to saturated fatty acids and increased omega‐3 consumption can help to manage anorexia nervosa and bulimia nervosa (Leone et al., 2018).

Inflammatory processes as well as oxidative stress and mitochondrial dysfunction are also linked to the pathogenesis of bipolar disorder. As a result, adherence to the Mediterranean diet may serve as a protective factor in this Axis I disorder through reversing these conditions (Łojko et al., 2018). Mediterranean diet can be suggested in schizophrenia as well. Comorbidities, such as obesity, cardiovascular diseases, and metabolic syndrome, are usually present along with schizophrenia. Furthermore, some medications used in this mental disorder can induce weight gain. Mediterranean diet may prevent weight gain, promote weight loss, and ameliorate lipid profile, hypertension, and insulin resistance (components of metabolic syndrome) in schizophrenic patients (Groszewska et al., 2015).

As mentioned earlier, more than two‐thirds of the studies in the present review observed protective associations between the Mediterranean diet and Axis I disorders. However, some studies did not achieve significant results, and even two studies found a direct relationship between receiving Mediterranean diet and increasing these disorders. One was a cross‐sectional study (Vassou et al., 2021) that found positive correlation between Mediterranean diet and depression. However, the correlation between anxiety and the dietary pattern was negative. The justification of this study for the unexpected finding was that because depression is usually more severe and discernable than anxiety, depressive patients may promptly seek therapeutic strategies including adopting a healthy diet and change their nutritional habits. The other study was also cross‐sectional (Carlos et al., 2020) and found a direct relationship between adherence to Mediterranean diet and trait‐anxiety. The study explained that this result could have been due to an association between obsession with healthy eating and anxiety problems.

So far, a number of review studies have been conducted on Mediterranean diet and mental disorders. For instance, a previous systematic review and meta‐analysis carried out on dietary indices and depressive outcomes found protective relationship between adherence to Mediterranean diet and reducing depression (Lassale et al., 2019). In another review study, although an inverse relationship was found between receiving Mediterranean diet and depression in the included cross‐sectional studies, the result of cohort studies showed no significant relationship (Shafiei et al., 2019). In addition, a meta‐analysis revealed a significant relationship between receiving a healthy dietary pattern (such as Mediterranean diet) and reduced symptoms of ADHD (Del‐Ponte et al., 2019). A systematic review has also evaluated the short‐term effects of the Mediterranean‐style dietary pattern on anxiety, cognition, and mood and implied that this dietary pattern could be an encouraging approach for improving mental well‐being (Esgunoglu et al., 2021). Besides, a narrative review has pointed to the supportive role of Mediterranean diet in patients with bipolar disorder. High consumption of sugar, fat, and carbohydrate, which are common among these patients, may negatively affect mental health, whereas Mediterranean diet with a high content of fruits, vegetables, and fish can reduce inflammation and oxidative stress in bipolar patients (Beyer & Payne, 2016).

Among the strengths of this study are the novelty of this topic and the inclusion of most mental disorders in a single study and also a detailed search in the form of a systematic review. However, the present review has some limitations. For instance, the time span of the literature search was limited to 5 years, only two databases were included, and no meta‐analysis was performed on the data. Another limitation was that substance use was not included as an Axis I disorder in this systematic review. Because this disorder is somewhat different to other disorders in terms of direct contact with external physical stimulants, and also because so many drugs and solvents can be categorized into this group (Nutt et al., 2007) which make it difficult to do a comprehensive search.

It is suggested that in the future, more review studies be conducted with a wider time span of literature search, on various diets, and on different age groups (including children) to examine the diets' associations with Axis I disorders. Conducting a systematic review or meta‐analysis in the field of interventional studies can also have a significant impact on controlling and preventing these disorders.

## 5 CONCLUSION

It seems that the Mediterranean diet can be a practical diet in the control of Axis I disorders (especially depression and anxiety) and can have protective effects in this regard. Therefore, patients with Axis I disorders, including those who are not suitable for pharmacotherapy or psychotherapy, can utilize this diet as an alternative or complementary treatment. Future systematic reviews should focus on understanding the efficacy of different dietary patterns on these disorders. There is also a fundamental need to better educate individuals and physicians with the role of diets and nutrients in maintaining mental health.

## CONFLICT OF INTEREST

The authors declare no conflict of interest.

## ETHICAL APPROVAL

This study does not involve any human or animal testing.

## APPENDIX A

### Search strategies in PubMed and Scopus databases

#### PubMed

("Depressive symptoms"[Title/Abstract] OR "Depressive symptom"[Title/Abstract] OR “Emotional Depression” [Title/Abstract] OR (Depression [Title/Abstract] AND Emotional [Title/Abstract]) OR “Depressive Disorders” [Title/Abstract] OR (Depression [Title/Abstract] AND Endogenous [Title/Abstract]) OR “Endogenous Depression” [Title/Abstract] OR (Depression[Title/Abstract] AND Neurotic [Title/Abstract]) OR “Neurotic Depression” [Title/Abstract] OR “Unipolar Depression” [Title/Abstract] OR (Depression [Title/Abstract] AND Unipolar [Title/Abstract]) OR Depression[Title/Abstract] OR "Depressive disorder*"[Title/Abstract] OR (Symptom [Title/Abstract] AND Depressive [Title/Abstract]) OR (Symptoms [Title/Abstract] AND Depressive[Title/Abstract]) OR (Disorder [Title/Abstract] AND Depressive[Title/Abstract]) OR (Disorders[Title/Abstract] AND Depressive[Title/Abstract]) OR (Neurosis [Title/Abstract] AND Depressive[Title/Abstract]) OR "Depressive Neuroses" [Title/Abstract] OR "Depressive Neurosis"[Title/Abstract] OR (Neuroses[Title/Abstract] AND Depressive[Title/Abstract]) OR (Depressions[Title/Abstract] AND Endogenous[Title/Abstract]) OR "Endogenous Depressions"[Title/Abstract] OR "Depressive Syndrome"[Title/Abstract] OR" Depressive Syndromes"[Title/Abstract] OR (Syndrome [Title/Abstract] AND Depressive[Title/Abstract]) OR (Syndromes[Title/Abstract] AND Depressive[Title/Abstract]) OR (Depressions[Title/Abstract] AND Neurotic[Title/Abstract]) OR" Neurotic Depressions"[Title/Abstract] OR Melancholia [Title/Abstract] OR Melancholias[Title/Abstract] OR (Depressions[Title/Abstract] AND Unipolar[Title/Abstract]) OR "Unipolar Depressions"[Title/Abstract] OR (Disorder[Title/Abstract] AND Bipolar[Title/Abstract]) OR "Bipolar Mood Disorder"[Title/Abstract] OR "Bipolar disorder*"[Title/Abstract] OR “Bipolar Disorders” [Title/Abstract] OR "Bipolar Mood Disorders"[Title/Abstract] OR (Disorder[Title/Abstract] AND "Bipolar Mood"[Title/Abstract]) OR ("Mood Disorder"[Title/Abstract] AND Bipolar[Title/Abstract]) OR “Bipolar Disorders” [Title/Abstract] OR (“Affective Psychosis” [Title/Abstract] AND Bipolar [Title/Abstract]) OR “Bipolar Affective Psychosis” [Title/Abstract] OR (Psychoses [Title/Abstract] AND “Bipolar Affective” [Title/Abstract]) OR (Psychosis [Title/Abstract] AND “Bipolar Affective” [Title/Abstract]) OR “Manic‐Depressive Psychosis” [Title/Abstract] OR “Manic Depressive Psychosis” [Title/Abstract] OR (Psychosis [Title/Abstract] AND “Manic‐Depressive” [Title/Abstract]) OR (Psychosis [Title/Abstract] AND “Manic Depressive” [Title/Abstract]) OR (Psychoses [Title/Abstract] AND “Manic‐Depressive” [Title/Abstract]) OR (Psychoses [Title/Abstract] AND “Manic Depressive” [Title/Abstract]) OR ("Mood Disorder"[Title/Abstract] AND Bipolar[Title/Abstract]) OR (Depression[Title/Abstract] AND Bipolar[Title/Abstract]) OR "Bipolar Depression"[Title/Abstract] OR "Manic Depression"[Title/Abstract] OR (Depression[Title/Abstract] AND Manic[Title/Abstract]) OR (Depressions[Title/Abstract] AND Manic[Title/Abstract]) OR "Manic Disorder"[Title/Abstract] OR (Disorder[Title/Abstract] AND Manic[Title/Abstract]) OR "Manic Disorders"[Title/Abstract] OR Angst[Title/Abstract] OR (Anxieties[Title/Abstract] AND Social[Title/Abstract]) OR "Social Anxieties"[Title/Abstract] OR "Generalized anxiety disorder*"[Title/Abstract] OR "anxiety disorder*"[Title/Abstract] OR anxiety[Title/Abstract] OR “Social Anxiety” [Title/Abstract] OR (Anxiety [Title/Abstract] AND Social [Title/Abstract]) OR “Anxiety Disorder” [Title/Abstract] OR (Disorders[Title/Abstract] AND Anxiety [Title/Abstract]) OR (Neuroses[Title/Abstract] AND Anxiety [Title/Abstract]) OR “Anxiety Neuroses” [Title/Abstract] OR (“Anxiety States” [Title/Abstract] AND Neurotic [Title/Abstract]) OR “Neurotic Anxiety State” [Title/Abstract] OR “Neurotic Anxiety States” [Title/Abstract] OR (State [Title/Abstract] AND “ Neurotic Anxiety” [Title/Abstract]) OR (States [Title/Abstract] AND “Neurotic Anxiety” [Title/Abstract]) OR Hypervigilance[Title/Abstract] OR Nervousness[Title/Abstract] OR Anxiousness[Title/Abstract] OR Schizophrenia[Title/Abstract] OR Schizophrenias[Title/Abstract] OR "Schizophrenic Disorders"[Title/Abstract] OR (Disorder[Title/Abstract] AND Schizophrenic[Title/Abstract]) OR "Schizophrenic Disorder"[Title/Abstract] OR (Disorder[Title/Abstract] AND "Obsessive‐Compulsive" [Title/Abstract]) OR OCD[Title/Abstract] OR "obsessive‐compulsive symptom*"[Title/Abstract] OR (Disorders[Title/Abstract] AND "Obsessive‐Compulsive" [Title/Abstract]) OR "Obsessive Compulsive Disorder"[Title/Abstract] OR "Obsessive‐Compulsive Disorders"[Title/Abstract] OR (Neurosis[Title/Abstract] AND "Obsessive‐Compulsive"[Title/Abstract]) OR (Neuroses[Title/Abstract] AND "Obsessive‐Compulsive"[Title/Abstract]) OR (Neurosis[Title/Abstract] AND "Obsessive Compulsive"[Title/Abstract]) OR ("Obsessive‐Compulsive" [Title/Abstract] AND Neuroses[Title/Abstract]) OR ("Obsessive‐Compulsive" [Title/Abstract] AND Neurosis[Title/Abstract]) OR "Anankastic Personality"[Title/Abstract] OR "Anankastic Personalities"[Title/Abstract] OR "Anankastic Personalities"[Title/Abstract] OR (Personalities[Title/Abstract] AND Anankastic[Title/Abstract]) OR (Personality[Title/Abstract] AND Anankastic[Title/Abstract]) OR ADHD[Title/Abstract] OR "attention‐deficit/hyperactivity disorder*"[Title/Abstract] OR "ADHD/ Hyperactivity"[Title/Abstract] OR "Attention Deficit Hyperactivity Disorder"[Title/Abstract] OR "Attention Deficit‐Hyperactivity Disorder"[Title/Abstract] OR "Attention Deficit‐Hyperactivity Disorders"[Title/Abstract] OR ("Deficit‐Hyperactivity Disorder"[Title/Abstract] AND Attention[Title/Abstract]) OR ("Deficit‐Hyperactivity Disorders"[Title/Abstract] AND Attention[Title/Abstract]) OR ("Deficit‐Hyperactivity Disorders"[Title/Abstract] AND Attention[Title/Abstract]) OR "Post‐traumatic stress disorder*"[Title/Abstract] OR PTSD[Title/Abstract] OR "post‐traumatic"[Title/Abstract] OR anorexia[Title/Abstract] OR "eating disorder*"[Title/Abstract] OR Anorexia nervosa[Title/Abstract] OR "Binge eating"[Title/Abstract] OR "Bulimia nervosa"[Title/Abstract] OR "binge‐eating disorder*"[Title/Abstract] OR "Anorexia Nervosas"[Title/Abstract] OR (Nervosa[Title/Abstract] AND Anorexia[Title/Abstract]) OR (Nervosas[Title/Abstract] AND Anorexia[Title/Abstract]) OR (Nervosa[Title/Abstract] AND Bulimia[Title/Abstract]) OR bulimia[Title/Abstract] OR “Eating Disorders” [Title/Abstract] OR “Eating Disorder” [Title/Abstract] OR" Binge‐Eating Disorders"[Title/Abstract] OR (Disorder[Title/Abstract] AND “Binge‐Eating”[Title/Abstract]) OR (Disorders[Title/Abstract] AND “Binge‐Eating”[Title/Abstract]) OR “Psychotic Disorder*” [Title/Abstract] OR Psychotic [Title/Abstract] OR Psychosis [Title/Abstract] OR Psychoses [Title/Abstract]) AND ("Mediterranean Diet"[Title/Abstract] OR Mediterran*[Title/Abstract] OR “Post‐Traumatic Neuroses”[Title/Abstract] OR “Posttraumatic Neuroses”[Title/Abstract] OR “Post‐Traumatic Stress Disorders”[Title/Abstract] OR “Post Traumatic Stress Disorders”[Title/Abstract] OR “Posttraumatic Stress Disorders”[Title/Abstract] OR “Posttraumatic Stress Disorder”[Title/Abstract] OR “Post Traumatic Stress Disorder”[Title/Abstract] OR “Delayed Onset Post‐Traumatic Stress Disorder”[Title/Abstract] OR “Delayed Onset Post Traumatic Stress Disorder”[Title/Abstract] OR “Chronic Post‐Traumatic Stress Disorder”[Title/Abstract] OR “Chronic Post Traumatic Stress Disorder”[Title/Abstract] OR “Moral Injury”[Title/Abstract] OR “Moral Injuries”[Title/Abstract] OR “Acute Post‐Traumatic Stress Disorder”[Title/Abstract] OR “Acute Post Traumatic Stress Disorder” [Title/Abstract]) AND ("Mediterranean Diet” [Title/Abstract] OR (Diets [Title/Abstract] AND Mediterranean [Title/Abstract]) OR "Mediterranean Diets"[Title/Abstract] OR Mediterran*[Title/Abstract]) AND 2016/01/01:2021/06/18[dp]

#### Scopus

(TITLE‐ABS‐KEY ("Depressive Symptoms") OR TITLE‐ABS‐KEY ("Depressive Symptom") OR TITLE‐ABS‐KEY(Depression) OR TITLE‐ABS‐KEY ("Depressive disorder*") OR TITLE‐ABS‐KEY ("Emotional Depression") OR (TITLE‐ABS‐KEY (Depression) AND TITLE‐ABS‐KEY (Emotional)) OR TITLE‐ABS‐KEY ("Depressive Disorders") OR (TITLE‐ABS‐KEY (Depression) AND TITLE‐ABS‐KEY (Endogenous)) OR TITLE‐ABS‐KEY ("Endogenous Depression") OR (TITLE‐ABS‐KEY (Depression) AND TITLE‐ABS‐KEY (Neurotic)) OR TITLE‐ABS‐KEY ("Neurotic Depression") OR TITLE‐ABS‐KEY ("Unipolar Depression") OR (TITLE‐ABS‐KEY (Depression) AND TITLE‐ABS‐KEY (Unipolar)) OR (TITLE‐ABS‐KEY (Symptom) AND TITLE‐ABS‐KEY (Depressive)) OR (TITLE‐ABS‐KEY (Symptoms) AND TITLE‐ABS‐KEY (Depressive)) OR (TITLE‐ABS‐KEY (Disorder) AND TITLE‐ABS‐KEY (Depressive)) OR (TITLE‐ABS‐KEY (Disorders) AND TITLE‐ABS‐KEY (Depressive)) OR (TITLE‐ABS‐KEY (Neurosis) AND TITLE‐ABS‐KEY (Depressive)) OR TITLE‐ABS‐KEY ("Depressive Neuroses") OR TITLE‐ABS‐KEY ("Depressive Neurosis") OR (TITLE‐ABS‐KEY (Neuroses) AND TITLE‐ABS‐KEY (Depressive)) OR (TITLE‐ABS‐KEY (Depressions) AND TITLE‐ABS‐KEY (Endogenous)) OR TITLE‐ABS‐KEY("Endogenous Depressions") OR TITLE‐ABS‐KEY("Depressive Syndrome") OR TITLE‐ABS‐KEY("Depressive Syndromes") OR (TITLE‐ABS‐KEY (Syndrome) AND TITLE‐ABS‐KEY (Depressive)) OR (TITLE‐ABS‐KEY (Syndromes) AND TITLE‐ABS‐KEY (Depressive)) OR (TITLE‐ABS‐KEY (Depressions) AND TITLE‐ABS‐KEY (Neurotic)) OR TITLE‐ABS‐KEY("Neurotic Depressions") OR TITLE‐ABS‐KEY(Melancholia) OR TITLE‐ABS‐KEY(Melancholias) OR (TITLE‐ABS‐KEY (Depressions) AND TITLE‐ABS‐KEY (Unipolar)) OR TITLE‐ABS‐KEY("Unipolar Depressions") OR (TITLE‐ABS‐KEY (Disorder) AND TITLE‐ABS‐KEY (Bipolar)) OR TITLE‐ABS‐KEY("Bipolar disorder*") OR TITLE‐ABS‐KEY("Bipolar Mood Disorder") OR TITLE‐ABS‐KEY("Bipolar Mood Disorder") OR (TITLE‐ABS‐KEY (Disorder) AND TITLE‐ABS‐KEY (“Bipolar Mood”)) OR (TITLE‐ABS‐KEY (“Mood Disorder”) AND TITLE‐ABS‐KEY (Bipolar)) OR (TITLE‐ABS‐KEY (Depression) AND TITLE‐ABS‐KEY (Bipolar)) OR TITLE‐ABS‐KEY("Bipolar Depression") OR TITLE‐ABS‐KEY("Manic Depression") OR (TITLE‐ABS‐KEY (Depression) AND TITLE‐ABS‐KEY (Manic)) OR (TITLE‐ABS‐KEY (Depressions) AND TITLE‐ABS‐KEY (Manic)) OR TITLE‐ABS‐KEY("Manic Disorder") OR (TITLE‐ABS‐KEY (Disorder) AND TITLE‐ABS‐KEY (Manic)) OR TITLE‐ABS‐KEY(Angst) OR TITLE‐ABS‐KEY ("Bipolar Disorders") OR (TITLE‐ABS‐KEY ("Affective Psychosis") AND TITLE‐ABS‐KEY (Bipolar)) OR TITLE‐ABS‐KEY ("Bipolar Affective Psychosis") OR (TITLE‐ABS‐KEY (Psychoses) AND TITLE‐ABS‐KEY ("Bipolar Affective")) OR (TITLE‐ABS‐KEY (Psychosis) AND TITLE‐ABS‐KEY ("Bipolar Affective")) OR TITLE‐ABS‐KEY ("Manic‐Depressive Psychosis") OR TITLE‐ABS‐KEY ("Manic Depressive Psychosis") OR (TITLE‐ABS‐KEY (Psychosis) AND TITLE‐ABS‐KEY ("Manic‐Depressive")) OR (TITLE‐ABS‐KEY (Psychosis) AND TITLE‐ABS‐KEY ("Manic Depressive")) OR (TITLE‐ABS‐KEY (Psychoses) AND TITLE‐ABS‐KEY ("Manic‐Depressive")) OR (TITLE‐ABS‐KEY (Psychoses) AND TITLE‐ABS‐KEY ("Manic Depressive")) OR TITLE‐ABS‐KEY("Social Anxiety") OR (TITLE‐ABS‐KEY (Anxiety) AND TITLE‐ABS‐KEY (Social)) OR TITLE‐ABS‐KEY("Anxiety Disorder") OR (TITLE‐ABS‐KEY (Disorder) AND TITLE‐ABS‐KEY (Anxiety)) OR (TITLE‐ABS‐KEY (Disorders) AND TITLE‐ABS‐KEY (Anxiety)) OR (TITLE‐ABS‐KEY (Neuroses) AND TITLE‐ABS‐KEY (Anxiety)) OR TITLE‐ABS‐KEY("Anxiety Neuroses") OR (TITLE‐ABS‐KEY ("Anxiety States") AND TITLE‐ABS‐KEY (Neurotic)) OR (TITLE‐ABS‐KEY ("Anxiety State") AND TITLE‐ABS‐KEY (Neurotic)) OR TITLE‐ABS‐KEY("Neurotic Anxiety State") OR TITLE‐ABS‐KEY("Neurotic Anxiety States") OR (TITLE‐ABS‐KEY (State) AND TITLE‐ABS‐KEY ("Neurotic Anxiety")) OR (TITLE‐ABS‐KEY (States) AND TITLE‐ABS‐KEY ("Neurotic Anxiety")) OR TITLE‐ABS‐KEY("Generalized anxiety disorder*") OR TITLE‐ABS‐KEY("anxiety disorder*") OR TITLE‐ABS‐KEY(anxiety) OR (TITLE‐ABS‐KEY (Anxieties) AND TITLE‐ABS‐KEY (Social)) OR TITLE‐ABS‐KEY("Social Anxieties") OR TITLE‐ABS‐KEY(Hypervigilance)OR TITLE‐ABS‐KEY(Nervousness) OR TITLE‐ABS‐KEY(Anxiousness) OR TITLE‐ABS‐KEY(Schizophrenia) OR TITLE‐ABS‐KEY (Schizophrenias) OR TITLE‐ABS‐KEY("Schizophrenic Disorders") OR (TITLE‐ABS‐KEY (Disorder) AND TITLE‐ABS‐KEY (Schizophrenic)) OR (TITLE‐ABS‐KEY (Disorders) AND TITLE‐ABS‐KEY (Schizophrenic)) OR TITLE‐ABS‐KEY("Schizophrenic Disorder") OR (TITLE‐ABS‐KEY (Disorder) AND TITLE‐ABS‐KEY ("Obsessive‐Compulsive")) OR TITLE‐ABS‐KEY(OCD) OR TITLE‐ABS‐KEY ("obsessive‐compulsive symptom*") OR (TITLE‐ABS‐KEY (Disorders) AND TITLE‐ABS‐KEY (("Obsessive‐Compulsive")) OR TITLE‐ABS‐KEY ("Obsessive Compulsive Disorder") OR TITLE‐ABS‐KEY ("Obsessive‐Compulsive Disorders") OR (TITLE‐ABS‐KEY (Neurosis) AND TITLE‐ABS‐KEY (" Obsessive‐Compulsive")) OR (TITLE‐ABS‐KEY (Neuroses) AND TITLE‐ABS‐KEY ("Obsessive‐Compulsive")) OR (TITLE‐ABS‐KEY (Neurosis) AND TITLE‐ABS‐KEY (" Obsessive Compulsive")) OR TITLE‐ABS‐KEY("Obsessive‐Compulsive Neuroses") OR TITLE‐ABS‐KEY("Obsessive‐Compulsive Neurosis") OR TITLE‐ABS‐KEY("Anankastic Personality") OR TITLE‐ABS‐KEY("Anankastic Personalities") OR (TITLE‐ABS‐KEY (Personalities) AND TITLE‐ABS‐KEY (Anankastic)) OR (TITLE‐ABS‐KEY (Personality) AND TITLE‐ABS‐KEY (Anankastic)) OR TITLE‐ABS‐KEY("Attention Deficit Hyperactivity Disorder") OR TITLE‐ABS‐KEY(ADHD) OR TITLE‐ABS‐KEY("attention‐deficit/hyperactivity disorder*") OR TITLE‐ABS‐KEY("ADHD/ Hyperactivity") OR TITLE‐ABS‐KEY("Attention Deficit‐Hyperactivity Disorder") OR TITLE‐ABS‐KEY("Attention Deficit‐Hyperactivity Disorders") OR (TITLE‐ABS‐KEY (" Deficit‐Hyperactivity Disorder") AND TITLE‐ABS‐KEY (Attention)) OR (TITLE‐ABS‐KEY (" Deficit‐Hyperactivity Disorders") AND TITLE‐ABS‐KEY (Attention)) OR TITLE‐ABS‐KEY("Anorexia Nervosas") OR (TITLE‐ABS‐KEY (Nervosa) AND TITLE‐ABS‐KEY (Anorexia)) OR (TITLE‐ABS‐KEY (Nervosas) AND TITLE‐ABS‐KEY (Anorexia)) OR (TITLE‐ABS‐KEY (Nervosa) AND TITLE‐ABS‐KEY (Bulimia)) OR TITLE‐ABS‐KEY(bulimia) OR TITLE‐ABS‐KEY("eating disorder*") OR TITLE‐ABS‐KEY(Anorexia nervosa) OR TITLE‐ABS‐KEY("Binge eating") OR TITLE‐ABS‐KEY("Bulimia nervosa") OR TITLE‐ABS‐KEY("binge‐eating disorder*") OR TITLE‐ABS‐KEY ("Eating Disorders") OR TITLE‐ABS‐KEY ("Eating Disorder") OR TITLE‐ABS‐KEY("Binge Eating Disorder") OR TITLE‐ABS‐KEY("Binge‐Eating Disorders") OR (TITLE‐ABS‐KEY (Disorder) AND TITLE‐ABS‐KEY (" Binge‐Eating")) OR (TITLE‐ABS‐KEY (Disorders) AND TITLE‐ABS‐KEY ("Binge‐Eating")) OR TITLE‐ABS‐KEY("Post‐traumatic stress disorder*") OR TITLE‐ABS‐KEY(PTSD) OR TITLE‐ABS‐KEY("post‐traumatic") OR TITLE‐ABS‐KEY(anorexia) OR TITLE‐ABS‐KEY (“Psychotic Disorder*”) OR TITLE‐ABS‐KEY(Psychotic) OR TITLE‐ABS‐KEY(Psychosis) OR TITLE‐ABS‐KEY(Psychoses) OR TITLE‐ABS‐KEY (“Post‐Traumatic Neuroses”) OR TITLE‐ABS‐KEY (“Posttraumatic Neuroses”) OR TITLE‐ABS‐KEY (“Post‐Traumatic Stress Disorders”) OR TITLE‐ABS‐KEY (“Post Traumatic Stress Disorders”) OR TITLE‐ABS‐KEY (“Posttraumatic Stress Disorders”) OR TITLE‐ABS‐KEY (“Posttraumatic Stress Disorder”) OR TITLE‐ABS‐KEY (“Post Traumatic Stress Disorder”) OR TITLE‐ABS‐KEY (“Delayed Onset Post‐Traumatic Stress Disorder”) OR TITLE‐ABS‐KEY (“Delayed Onset Post Traumatic Stress Disorder”) OR TITLE‐ABS‐KEY (“Chronic Post‐Traumatic Stress Disorder”) OR TITLE‐ABS‐KEY (“Chronic Post Traumatic Stress Disorder”) OR TITLE‐ABS‐KEY (“Moral Injury”) OR TITLE‐ABS‐KEY (“Moral Injuries”) OR TITLE‐ABS‐KEY (“Acute Post‐Traumatic Stress Disorder”) OR TITLE‐ABS‐KEY (“Acute Post Traumatic Stress Disorder”)) AND (TITLE‐ABS‐KEY(Diets) AND TITLE‐ABS‐KEY(Mediterranean)) OR TITLE‐ABS‐KEY("Mediterranean Diets") OR TITLE‐ABS‐KEY ("Mediterranean Diet”) OR TITLE‐ABS‐KEY(Mediterran*)) AND ((PUBYEAR > 2015 AND PUBYEAR < 2021) OR PUBDATETXT("January 2021" OR "February 2021" OR "March 2021" OR "April 2021" OR "May 2021" OR "June 2021"))

## APPENDIX B

### Quality assessment of cohort studies exploring the association between the Mediterranean diet and Axis I disorders

**Table:**

	First author	year	Selection	Comparability	Outcome	Total quality
1	Cherian, L	2021	***	٭	٭٭	Good
2	Gianfredi, V	2021	**	٭٭	٭٭	Fair
3	Das, A	2021	***	٭٭	٭	Poor
4	Vall Castelló, J	2020	***	٭٭	٭٭	Good
5	Recchia, D	2020	***	٭٭	٭٭	Good
6	Winkens, L	2020	****	٭	٭	Poor
7	Fresán, U	2019	٭٭٭٭	٭٭	٭٭٭	Good
8	Ruiz‐Estigarribia, L	2019	٭٭٭٭	٭٭	٭٭	Good
9	Elstgeest, LEM	2019	***	٭٭	٭٭	Good
10	Leone, A	2018	٭٭٭	٭٭	٭٭	Good
11	Adjibade, M	2018	٭٭٭٭	٭٭	٭٭	Good
12	García‐Toro, M	2016	****	*	–	Poor
13	Notara, V	2016	***	*	**	Good
14	Sánchez‐Villegas, A	2016	****	٭٭	٭٭	Good
15	Lai, JS	2016	***	**	**	Good

Each star is equal to one score in each category of quality assessment.

### Quality assessment of cross‐sectional studies exploring the association between the Mediterranean diet and Axis I disorders

**Table:**

	First author	Year	Selection	Comparability	Outcome	Total quality
1	Mantzorou, M	2021	٭٭٭٭*	٭٭	٭٭	Good
2	Vassou, C	2021	٭٭٭٭٭	٭٭	٭٭٭	Good
3	Riera‐Sampol, A	2021	٭٭٭	٭٭	٭٭	Good
4	Sadeghi, O	2021	٭٭٭٭	٭٭	٭٭	Good
5	Kaufman‐Shriqui, V	2021	٭٭٭٭٭	–	٭٭	Poor
6	Trigueros, R	2020	٭٭٭٭	–	٭	Poor
7	Carlos, M	2020	٭٭٭	*	٭٭	Good
8	Açik, M	2020	٭*٭٭	٭٭	٭٭	Good
9	Gibson‐Smith, D	2020	٭٭٭٭٭	٭٭	٭٭٭	Good
10	Vicinanza, R	2020	٭٭	٭٭	٭	Poor
11	Paans, NPG	2019	**٭٭٭	٭٭	٭٭	Good
12	Mahdavi‐Roshan, M	2019	٭٭٭	٭*	٭٭	Good
13	Costa, R	2019	٭٭٭	–	**	Poor
14	Masana, MF	2019	٭٭٭	٭٭	٭٭	Good
15	Masana, MF	2018	٭٭٭	٭	٭٭	Good
16	Gibson‐Smith, D	2018	٭٭٭٭٭	٭٭	٭٭٭	Good
17	Pagliai, G	2018	٭٭٭	٭٭	٭٭	Good
18	Hernández‐Galiot, A	2017	٭٭٭	–	٭	Poor
19	Veronese, N	2016	*٭٭٭٭	٭٭	٭٭	Good

Each star is equal to one score in each category of quality assessment.

### Quality assessment of the case‐control studies exploring the association between the Mediterranean diet and Axis I disorders

**Table:**

	First author	Year	Selection	Comparability	Exposure	Total quality
1	Łojko, D	2019	٭٭٭	–	٭	Poor
2	Della Camera, PA	2017	٭	–	٭٭٭	Poor

Each star is equal to one score in each category of quality assessment.
